# A Treatise on Bright’s Disease of the Kidneys, Its Pathology, Diagnosis and Treatment

**Published:** 1884-11

**Authors:** 


A TREATISE ON BRIGHT'S DISEASE OF THE KIDNEYS, ITS PATHOLOGY, DIAGNOSIS AND TREATMENT. *With Chapters on the Anatomy of the Kidney, Albuminuria, and Urinary Secretions. By* HENRY B. MILLARD, M.D., A.M. *With numerous Original Illustrations. 8 vo., cloth, pp. xiv., 246. New York:* WILLIAM WOOD & CO. 1884.

A very modern book, which cannot be too highly recommended to medical students. Not incumbered with unnecessary details, not swollen by lengthy reports of cases, or the discussion of antiquated or doubtful modes of therapeutics, this manual would derive its best recommendations from its terseness and well selected material, were it not for the fact that it contains a large private experience on the part of the author, and this will give it the rank of a valuable contribution to a class of diseases, which, but lately considered incurable, now turn out full of promise for the methodical, scientific practitioner.

